# A Putative Association of a Single Nucleotide Polymorphism in *GPR126* with Aggressive Periodontitis in a Japanese Population

**DOI:** 10.1371/journal.pone.0160765

**Published:** 2016-08-10

**Authors:** Jirouta Kitagaki, Shizuka Miyauchi, Yoshihiro Asano, Atsuko Imai, Shinji Kawai, Ikumi Michikami, Motozo Yamashita, Satoru Yamada, Masahiro Kitamura, Shinya Murakami

**Affiliations:** 1 Department of Periodontology, Division of Oral Biology and Disease Control, Osaka University Graduate School of Dentistry, Suita, Osaka, Japan; 2 Department of Cardiovascular Medicine, Osaka University Graduate School of Medicine, Suita, Osaka, Japan; 3 Challenge to Intractable Oral Diseases, Center for Frontier Oral Science, Osaka University Graduate School of Dentistry, Suita, Osaka, Japan; Tokyo Ika Shika Daigaku, JAPAN

## Abstract

Periodontitis is an inflammatory disease causing loss of tooth-supporting periodontal tissue. Disease susceptibility to the rapidly progressive form of periodontitis, aggressive periodontitis (AgP), appears to be influenced by genetic risk factors. To identify these in a Japanese population, we performed whole exome sequencing of 41 unrelated generalized or localized AgP patients. We found that AgP is putatively associated with single nucleotide polymorphism (SNP) rs536714306 in the G-protein coupled receptor 126 gene, *GPR126* [c.3086 G>A (p.Arg1029Gln)]. Since GPR126 activates the cAMP/PKA signaling pathway, we performed cAMP ELISA analysis of cAMP concentrations, and found that rs536714306 impaired the signal transactivation of GPR126. Moreover, transfection of human periodontal ligament (HPDL) cells with wild-type or mutant GPR126 containing rs536714306 showed that wild-type GPR126 significantly increased the mRNA expression of bone sialoprotein, osteopontin, and Runx2 genes, while mutant GPR126 had no effect on the expression of these calcification-related genes. The increase in expression of these genes was through the GPR126-induced increase of bone morphogenic protein-2, inhibitor of DNA binding (ID) 2, and ID4 expression. These data indicate that GPR126 might be important in maintaining the homeostasis of periodontal ligament tissues through regulating the cytodifferentiation of HPDL cells. The *GPR126* SNP rs536714306 negatively influences this homeostasis, leading to the development of AgP, suggesting that it is a candidate genetic risk factor for AgP in the Japanese population.

## Introduction

Periodontitis is a complex inflammatory disease characterized by the destruction of tooth-supporting tissue (periodontal tissue) consisting of alveolar bone, periodontal ligament, and cementum [1]. Periodontitis is the major cause of tooth loss among adults aged over 40 years and its prevalence rates have been reported by the World Health Organization to be up to 20% worldwide [2–4]. Although the cause of the disease is bacterial biofilm, the onset and progress is influenced by a number of risk factors including age, systemic conditions, smoking, and genetic makeup [5].

Chronic periodontitis is the most common form of periodontitis, while aggressive periodontitis (AgP) is a more severe form and is characterized by rapid destruction of periodontal tissue at a young age in healthy individuals, leading to early tooth loss [6]. The AgP prevalence ranges from 0.2%–2.6% in Caucasians and African–Americans [7, 8], to 0.12%–0.47% in Chinese and 0.03% in Japanese populations [9, 10].

Several etiological studies have indicated the existence of high familial aggregation of AgP [9]. For example, familial studies have shown that its prevalence in affected siblings may reach at least 40–50% [11]. This indicates that genetic risk factors may be important to understand disease susceptibility, and many studies have been carried out to investigate the role of genetic polymorphisms in AgP. Polymorphisms in interleukin-1α -889 and interleukin-1β +3953 were first reported to be associated with the severity of AgP [12], while associations have been identified more recently in inflammatory cytokine and matrix metalloproteinase genes [13]. However, few genes have been established as AgP risk factors.

To identify genetic risk factors for a disease, detailed studies of associated candidate genes are required. However, the selection of candidate genes is dependent on prior knowledge about genes to be chosen [14]. Because of this limitation, it is essential to carry out the unbiased analysis of the entire genome to identify novel disease-associated genetic variants for AgP [15]. Such genome-wide association studies (GWAS) are comprehensive and hypothesis-free [16], and have recently identified genes not previously known to be involved in the etiology of the target disease such as hypertension [17] and diabetes [16, 18].

In the case of AgP, the intronic single nucleotide polymorphism (SNP) rs1537415 of the glycosyltransferase 6 domain containing 1 gene was previously reported to be associated with the disease in a German population [19]. The intronic SNP rs1333048 of the non-coding RNA cyclin dependent kinase inhibitor 2B antisense RNA 1 gene was also identified as the best shared genetic risk factor for coronary artery disease and AgP [20, 21]. A number of genetic risk factors for AgP have since been identified, including polymorphisms in genes encoding cyclooxygenase 2 [22], β-defensin 1 [23], and interleukin-10 [24]. Although several genetic risk factors for AgP have been identified in Caucasians, to date little is known for the Asian population.

Protein coding regions of genes make up only ~1% of the total human genomic DNA, but ~85% of disease-related genomic variants are located here or in canonical splice sites [25]. It is therefore important to sequence complete coding regions to understand rare human disease traits. Thus, to identify a genetic risk factor for AgP in a Japanese population, we conducted whole exome sequencing in this study and found that AgP is putatively associated with SNP rs536714306 of G-protein coupled receptor 126 gene (*GPR126*) [c.3086 G>A (p.Arg1029Gln)].

GPR126 belongs to the adhesion G-protein coupled receptor (GPCR) family and consists of an N-terminal extracellular domain, a seven transmembrane domain, and a C-terminal intracellular domain. The extracellular domain contains Complement, Uegf, Bmp1 (CUB), Pentraxin, and the GPCR-autoproteolysis inducing domain [26–28]. SNP rs536714306 is located between transmembrane V and VI.

We discovered that the SNP rs536714306 appears to impair transactivation of the GPR126 signal. We also found that GPR126 is associated with the cytodifferentiation of human periodontal ligament (HPDL) cells. These data imply that GPR126 is important in maintaining the homeostasis of periodontal ligament tissue.

## Materials and Methods

### Participants

Forty-one AgP patients from the Department of Periodontology, Osaka University Dental Hospital were recruited for this study. The patients included both those with localized AgP (n = 2) and those with generalized AgP (n = 39). Written informed consent was obtained from all the patients. This study was specifically approved by Osaka University Research Ethics Committee (No. 533).

### Clinical examination and phenotype definition

The definition of AgP was based on criteria from the American Academy of Periodontology [29]. The patients were diagnosed at the age of 18 to 39 years. AgP was classified as generalized or localized. Generalized AgP was defined as clinical attachment loss involving at least three teeth other than the first molars and incisors. Localized AgP was defined as clinical attachment loss involving at least two first molars or incisors. One of these teeth must be a first molar, and no more than two teeth except the first molars and incisors are affected by clinical attachment loss. We defined clinical attachment loss as >30% alveolar bone loss. The clinical parameters of the two populations were not significantly different with the exception of the type of AgP. Each subject underwent a thorough periodontal examination including probing pocket depth and bleeding on probing, which was measured at six sites around each tooth as shown in Table 1. The percentage of alveolar bone loss at each tooth was assessed by radiograph and the Schei ruler [30]. Additionally, the periodontal inflamed surface area (PISA) was calculated as described previously [31].

**Table 1:** Primer list for real-time PCR.

Gene	Primer sequence
HPRT	F: 5′-GGC AGT ATA ATC CAA AGA TGG TCA A-3′
	R: 5′-GTC AAG GGC ATA TCC TAC AAC AAA C-3′
Runx2	F: 5′-CAC TGG CGC TGC AAC AAG A-3′
	R: 5′-CAT TCC GGA GCT CAG CAG AAT AA-3′
BSP	F: 5′-CTG GCA CAG GGT ATA CAG GGT TAG-3′
	R: 5′-GCC TCT GTG CTG TTG GTA CTG GT-3′
osteopontin	F: 5′-ACA CAT ATG ATG GCC GAG GTG A-3′
	R: 5′-TGT GAG GTG ATG TCC TCG TCT GTA G-3′
BMP-2	F: 5′-CCT CTG GCT GAT CAT CTG AAC TC-3′
	R: 5′-GGG ACA CAG CAT GCC TTA GGA-3′
ID2	F: 5′-CAA AGC ACT GTG TGG CTG AAT AA-3′
	R: 5′-AAA GGT CCA TTC AAC TTG TCC TC-3′
ID4	F: 5′-ATG TCA CAT GTG CAC TGT TGG TTA G-3′
	R: 5′-AGG CTT AGG TAG GGT TCA GCA GTC-3′

F, forward; R, reverse

### Exome capture and sequencing

Exome capture and sequencing was completed by a commercial company (BGI Tech., Shenzhen, China). Genomic DNA was extracted from peripheral whole blood (Fujifilm, Tokyo, Japan), and exome capture was performed with Agilent SureSelect Human All Exon V4 or V5 kits (Agilent Technologies, Santa Clara, CA). Exome sequencing was undertaken with paired-end 100 or 101 base pair reads using Illumina HiSeq 2000 or 4000 platform (Illumina, Inc., San Diego, CA). The sequencing reads were aligned to the human reference genome (UCSC build HG19/HG18) using BWA software. Variants were called using GATK and SAMtools program pileup and annotated with the dbSNP database and 1000 Genomes Project database. The exome database is deposited in the DNA Data Bank of Japan (JGAS00000000024 and JGAS00000000040).

### Sanger sequencing

To confirm the identified variant sequence, Sanger sequencing was performed using *GPR126*-specific primers (forward: 5′-GAT TCA AGA TCC AGT CAT ATT TTA TGT G-3′, reverse: 5′-TAC CTC ATT TAT CTA TTC AAG ACC CTT T-3′) by a commercial service (Macrogen Japan, Tokyo, Japan). The sequence data were analyzed and compared with the human reference genome HG19.

### Construction of GPR126 vector

The full length human *GPR126* cDNA clone was provided by RIKEN BRC through the National Bio-Resource Project of the MEXT, Japan [32–35]. The construct was subcloned into pcDNA3.1(-) (Invitrogen, Carlsbad, CA) at *Not* I and *Kpn* I sites. A single point mutation was introduced into *GPR126* using the PrimeSTAR mutagenesis basal kit (TaKaRa, Shiga, Japan) and the primer set: forward: 5′-AGC AAC CAG ACC CTG AGA GAA GAA GT-3′, reverse: 5′-CAG GGT CTG GTT GCT TCT CTT GCC AT-3′ (the underlined bases represent the introduced mutation).

### Reagents and cell lines

The rabbit polyclonal antibody against GPR126 (ab75356) (diluted 1:500) was purchased from Abcam (Bristol, UK). The monoclonal antibody against β-Actin (diluted 1:3500) and Type IV collagen were purchased from Sigma (St Louis, MO). HEK293T cells were cultured in DMEM (Thermo Fisher Scientific, Carlsbad, CA) supplemented with 10% fetal calf serum (FCS) [36]. HPDL cells were purchased from ScienCell Research Laboraories (Carlsbad, CA) and were maintained in α-MEM (Nikken, Kyoto, Japan) supplemented with 10% FCS. To induce HPDL cell mineralization, we replaced this medium with a mineralization medium of α-MEM supplemented with 50 μg/mL L-ascorbic acid and 5 mM β-glycerophosphate.

### cAMP measurements

HEK293T or HPDL cells were transfected with empty vector or expression plasmids containing wild-type or mutant GPR126 constructs by Lipofectamine 3000 (Invitrogen). Cells were incubated with 3 μg/mL type IV collagen for 1 h, then lysed in 0.1 M HCl. Intracellular cAMP levels were analyzed by ELISA according to the manufacturer’s instructions (Enzo Life Sciences, Farmingdale, NY).

### Western blotting

Cells were lysed with RIPA buffer (Millipore, Temecula, CA) supplemented with Complete Mini (Roche, Mannheim, Germany) and centrifuged at 13,000 × *g* for 20 min. The resultant lysates were resolved by sodium dodecyl sulfate polyacrylamide gel electrophoresis, transferred to polyvinylidene difluoride membranes (Millipore), exposed to specific antibodies and horseradish peroxidase-conjugated secondary antibodies (GE Healthcare, Little Chalfont, UK), and visualized with chemiluminescence agents according to the manufacturer’s instructions [37].

### RNA isolation and real-time PCR

Total RNA of HPDL cells was isolated from using RNA bee (Tel-Test Inc. Friendswood, TX) and incubated with RNase-free DNase I (TaKaRa). cDNA synthesis was performed using a High Capacity RNA-to cDNA Kit (Applied Biosystems, Carlsbad, CA) according to the manufacturer’s instructions. Real-time PCR reaction was carried out using a 7300 Fast Real-Time RT-PCR system (Applied Biosystems) with Power PCR SYBR Master Mix (Applied Biosystems) and gene-specific PCR primers [37]. The primer sequences for the real-time PCR of hypoxanthine guanine phosphoribosyltransferase (HPRT), Runx2, BSP, osteopontin, BMP-2, ID2, and ID4 are shown in Table 1.

## Results

### Analysis of whole exome sequence

First, we examined the clinical characteristic of 41 unrelated AgP patients. The average of whole-mouth probing pocket depth, periodontal inflamed surface area (PISA), and alveolar bone loss was 4.22mm, 1358.94, and 37.53%, respectively (Table 2). Detailed information about affected teeth with at least 30% bone loss for each patient is listed in S1 Table. To identify a genetic risk factor for AgP in a Japanese population, we carried out whole exome sequencing of 41 AgP cases. The results were filtered to include the following variants: (1) variants with minor allele frequency (MAF) ≤0.01 in the 1000 Genome database, (2) variants with read depths ≥5, (3) variants not reported in dbSNP135, (4) non-synonymous variant predicted to be damaging by SIFT, (5) number of patients who possesses this variants ≥2, (6) variants with MAF ≤0.01 in the Human Genetic Variation Database (HGVD) as a control for Japanese genetic variation and giving a *P*-value ≤0.05 (chi-square test) after comparison with HGVD. The HGVD included 1208 Japanese individuals, indicating that the race characteristics of both the control and case populations were the same. All individuals in the HGVD had no clinical records associated with major disease. No additional information, including the periodontal status, about this population is available because of ethical issues [38, 39]. The mean coverage depth and coverage depth ≥10 was 145.14 and 98.34%, respectively. The mean overall coverage of the target and capture efficiency of the targets was 99.50 and 57.34%, respectively. The initial variants were filtered to a final 10 rare variants (Table 3).

**Table 2:** Clinical characteristic of individual subjects in the GWAS.

Subject Characteristics	
Age (yrs)	32.29 ± 6.82
Male/Female	14/27
Number of present teeth	27.98 ± 1.87
Probing pocket depth (mm)	4.22 ± 1.23
PISA	1358.94 ± 1067.72
Alveolar bone loss (%)	37.53 ± 15.54

PISA, periodontal inflamed surface area. Values represent means ± standard deviation.

**Table 3:** List of 10 filtered variants.

gene name	chromosome	position	*P*-value
THAP3	1	6688692	2.79 × 10^−2^
SLC1A4	2	65245299	2.20 × 10^−3^
ALSCR11	2	202357699	3.88 × 10^−2^
LAMB2	3	49161294	4.78 × 10^−2^
RASSF1	3	50378008	2.09 × 10^−2^
GPR126	6	142741092	2.20 × 10^−3^
FZD8	10	35928428	2.49 × 10^−11^
TAS2R31	12	11183447	2.54 × 10^−4^
PPP6R1	19	55758437	2.57 × 10^−4^
C22orf24	22	32330115	8.97 × 10^−6^

In a previous study, *GPR126* knockout mice were smaller than their wild-type littermates and showed abnormal joint formation [40], suggesting that GPR126 plays an important role in cytodifferentiation of hard-tissue–forming cells. Therefore, we focused on GPR126 for further experiments. We found that AgP is putatively associated with one of a novel SNP of *GPR126* [NM_001032394.2:c.3086 G>A (p.Arg1029Gln)], located on chromosome 6q24.1, with *P*-value 2.20 × 10^−3^, odds ratio 9.09, and 95% confidence interval: 1.64–50.36. The associated minor A allele frequency of the novel SNP demonstrated an enrichment of 2.17% in AgP cases (2.44% compared with 0.27% in HGVD controls) (Table 4). The collaborating institutions for the HGVD are Kyoto University, Yokohama City University, Tohoku University, The University of Tokyo, and the National Research Institute for Child Health and Development. However, genotyping information about *GPR126* SNP rs536714306 is only available from Kyoto University (300 subjects) and Yokohama City University (429 subjects). No information is available from the other three institutes regarding the absence of the minor allele of the SNP or the inadequacy of the read depth. Therefore, we used data from 729 subjects as the control group. Unfortunately, information about age, sex, and the periodontal status of HGVD controls is not available because of ethical issues, so the controls could not be age- or sex-matched with the AgP cases, and have an unknown periodontal status. The novel SNP is currently reported in dbSNP144 and registered as rs536714306. Sanger sequencing confirmed the SNP rs536714306 sequencing of 41 AgP cases. Because the *P*-value of 2.20 × 10^−3^ is insufficient to describe a genome-wide significance, we performed *in vitro* functional analysis to overcome this issue.

**Table 4:** GWAS findings.

Controls	G/G (%)	725 (99.45)
	G/A (%)	4 (0.55)
	A/A (%)	0 (0)
	MAF	0.27
Cases	G/G (%)	39 (95.12)
	G/A (%)	2 (4.88)
	A/A (%)	0 (0)
	MAF	2.44

MAF, minor allele frequency

### Effects of SNP rs536714306 on GPR126 signal transduction

**Fig 1:**
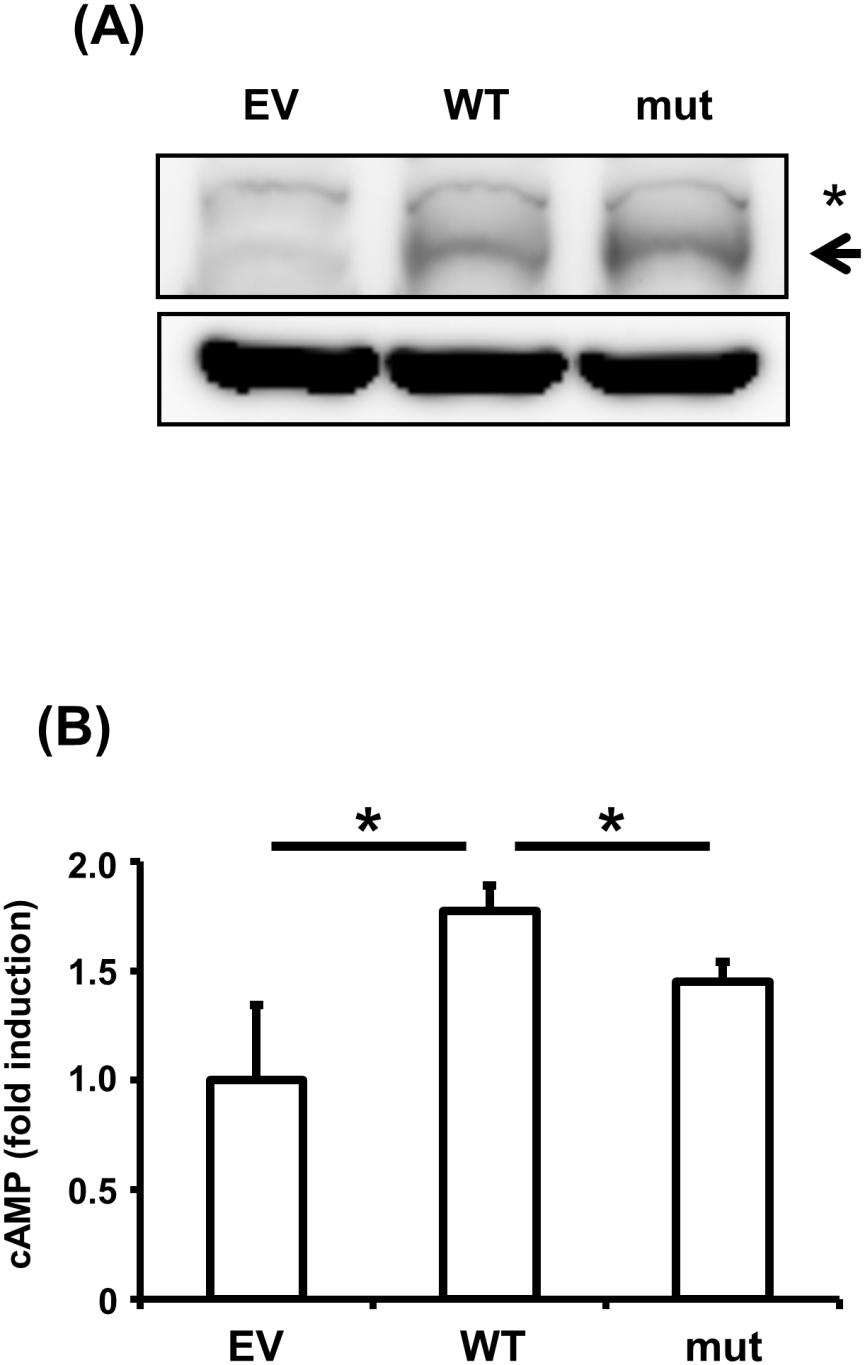
Effects of the SNP rs536714306 on *GPR126* signal transduction in HEK293T cells. (A) HEK293T cells were transfected with empty vector, wild-type GPR126, or mutant GPR126 containing SNP rs536714306 for 24 h, and expression of GPR126 and β-Actin was assessed by western blotting. Asterisk represents a non-specific band. (B) HEK293T cells were transfected with empty vector, wild-type GPR126, or mutant GPR126 for 48 h. Following a 1 h incubation with 3 μg/mL type IV collagen, cAMP expression was assessed by ELISA. Data represent the average and standard deviation of three independent experiments. *p <0.05. EV, empty vector; WT, wild-type GPR126; mut, mutant GPR126.

A number of studies have demonstrated that GPR126 elevates cAMP concentrations, activates protein kinase A (PKA), and increases the expression of several target genes [41]. To evaluate the effects of SNP rs536714306 on the GPR126 signal, we generated mutant GPR126 containing SNP rs536714306. Wild-type or mutant GPR126 was then introduced into HEK293T cells, and the expression of GPR126 and cAMP levels were assessed by western blotting and ELISA, respectively. GPR126 expression was clearly increased in HEK293T cells containing wild-type or mutant GPR126 (Fig 1A). However, while wild-type GPR126 significantly increased cAMP concentrations, mutant GPR126 had no effect on cAMP (Fig 1B). These data indicate that SNP rs536714306 impairs the GPR126 signal transactivation in HEK293T cells.

### Effects of SNP rs536714306 on the cytodifferentiation of HPDL cells

PDL tissue is a soft connective tissue interposed between the roots of the tooth and alveolar bone. It mechanically supports the teeth and plays nutritive and sensory roles to maintain the homeostasis of periodontal tissue [42, 43]. Because the PDL contains multipotential mesenchymal stem cells, PDL cells are able to differentiate into a number of hard tissue-forming cells, including osteoblasts and cementoblasts [44]. Therefore, when PDL cells are cultured in mineralization medium, the expression of calcification-related gene such as BSP is gradually increased [45].

**Fig 2:**
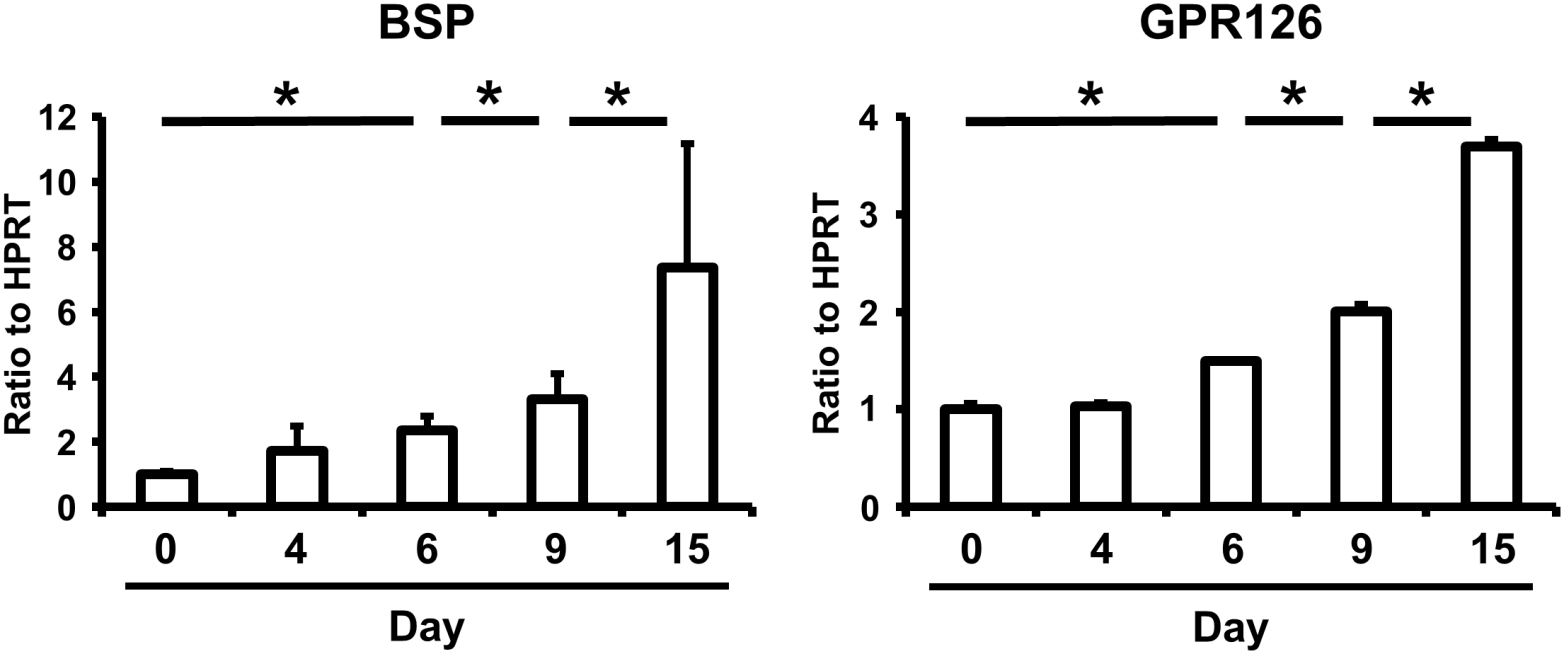
Expression of GPR126 during the cytodifferentiation of HPDL cells. HPDL cells were transfected with empty vector and incubated with mineralization medium for 4, 6, 9, and 15 days and the expression of BSP and GPR126 was assessed by real-time PCR. Data represent the average and standard deviation of four independent experiments. *p <0.05.

To analyze the possible association of GPR126 with the osteoblastic differentiation of HPDL cells, real-time PCR was performed using RNA from differentiated HPDL cells at each stage. We performed Sanger sequencing to confirm the rs536714306 genotype of the HPDL cells and found that the rs536714306 genotype of the HPDL was the wild type. As expected, the mRNA expression of *BSP* was increased during cell differentiation (Fig 2, left panel). *GPR126* mRNA expression was similarly increased during the cell differentiation (Fig 2, right panel), suggesting that GPR126 affects the cytodifferentiation of HPDL cells.

**Fig 3:**
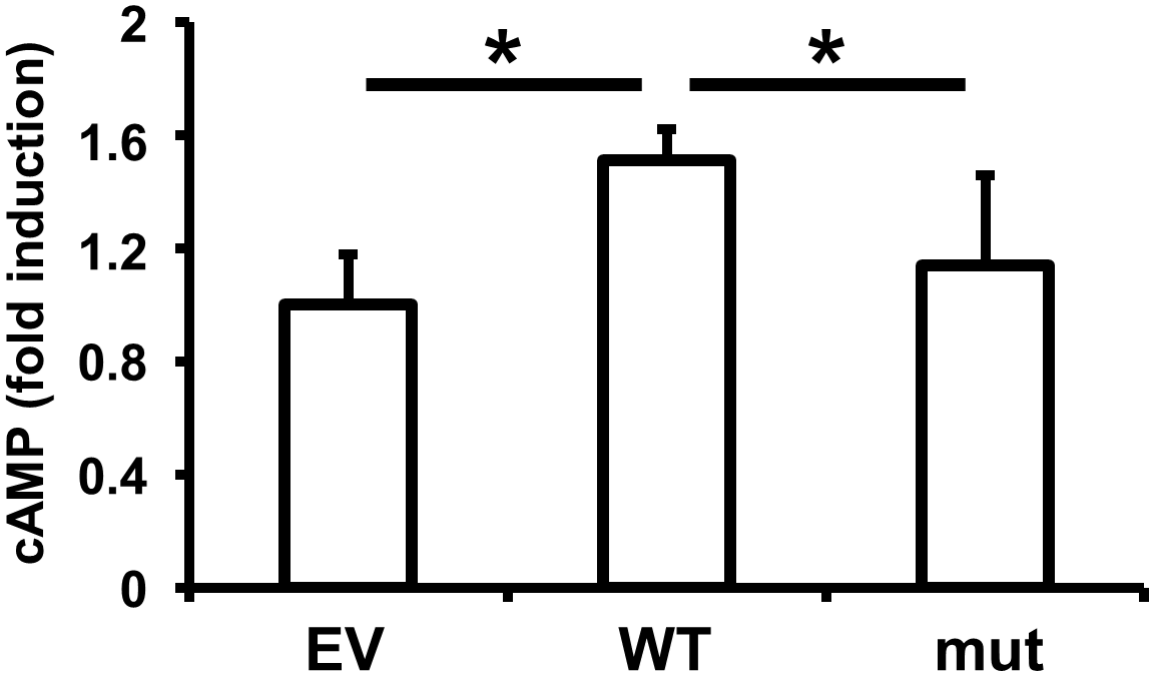
Effects of SNP rs536714306 on GPR126 signal transduction in HPDL cells. HPDL cells were transfected with empty vector, wild-type GPR126, or mutant GPR126 for 48 h. cAMP levels were assessed by ELISA following a 1 h incubation with 3 μg/mL type IV collagen. Data represent the average and standard deviation of three independent experiments. *p <0.05. EV, empty vector; WT, wild-type GPR126; mut, mutant GPR126.

Next, to evaluate the effects of SNP rs536714306 on GPR126 signal transactivation in HPDL cells, wild-type or mutant GPR126 containing SNP rs536714306 were introduced into HPDL cells. As assessed by ELISA, wild-type GPR126 increased cAMP concentrations in HPDL cells, but mutant GPR126 had no effect on cAMP (Fig 3). These data indicate that SNP rs536714306 impairs the GPR126 signal transactivation in HPDL cells.

**Fig 4:**
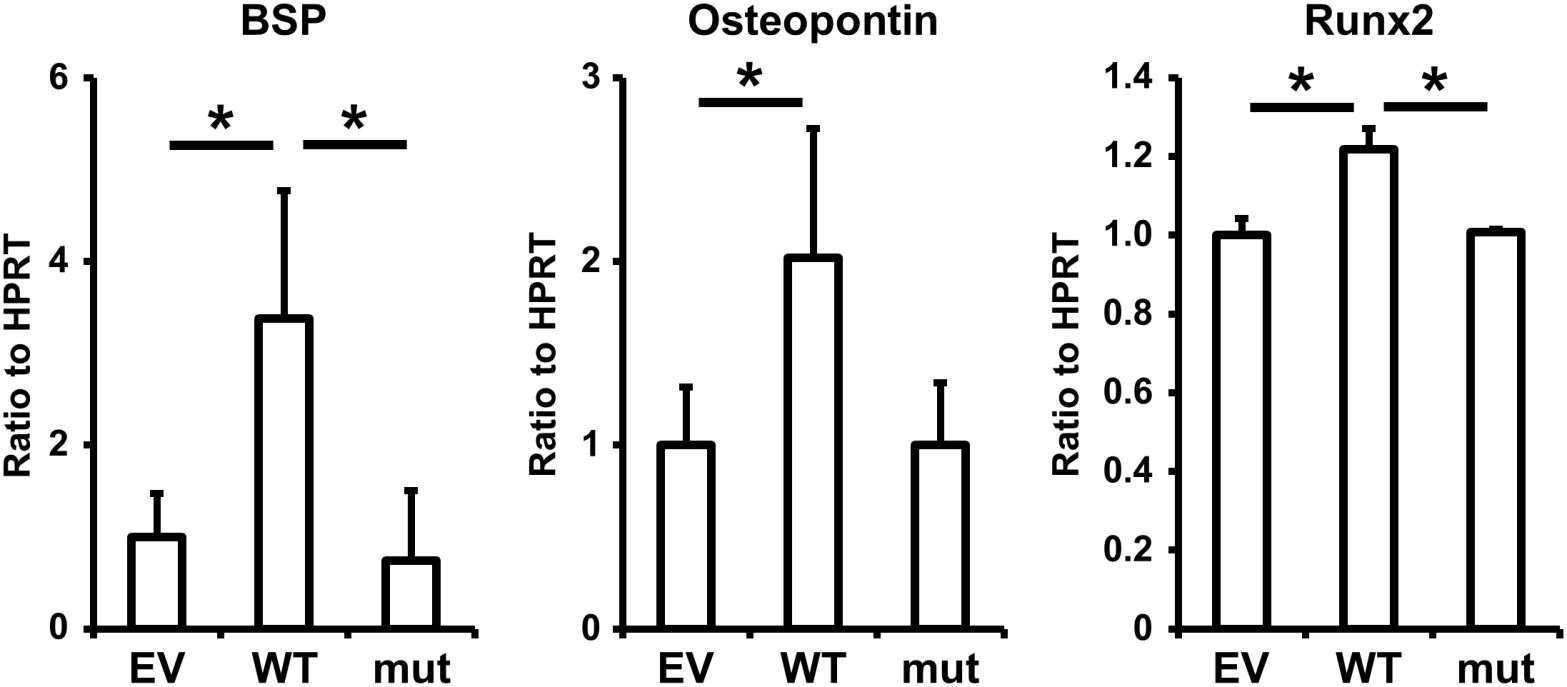
Effects of GPR126 on the cytodifferentiation of HPDL cells. HPDL cells were transfected with empty vector, wild-type GPR126, or mutant GPR126 for 96 h. The mRNA expression of BSP, osteopontin, and Runx2 was assessed by real-time PCR. Data represent the average and standard deviation of three independent experiments. *p <0.05. EV, empty vector; WT, wild-type GPR126; mut, mutant GPR126.

To investigate the effects of SNP rs536714306 on the cytodifferentiation of HPDL cells, wild-type or mutant GPR126 were transfected into HPDL cells. The mRNA expression of calcification-related genes such as BSP, osteopontin, and Runx2 was then assessed by real-time PCR. Wild-type, but not mutant, GPR126 significantly increased the mRNA expression of these genes (Fig 4). These data suggest that wild-type GPR126 up-regulates the cytodifferentiation of HPDL cells, but that the presence of SNP rs536714306 abrogates this effect.

### Mechanism of GPR126-induced cytodifferentiation of HPDL cells

**Fig 5:**
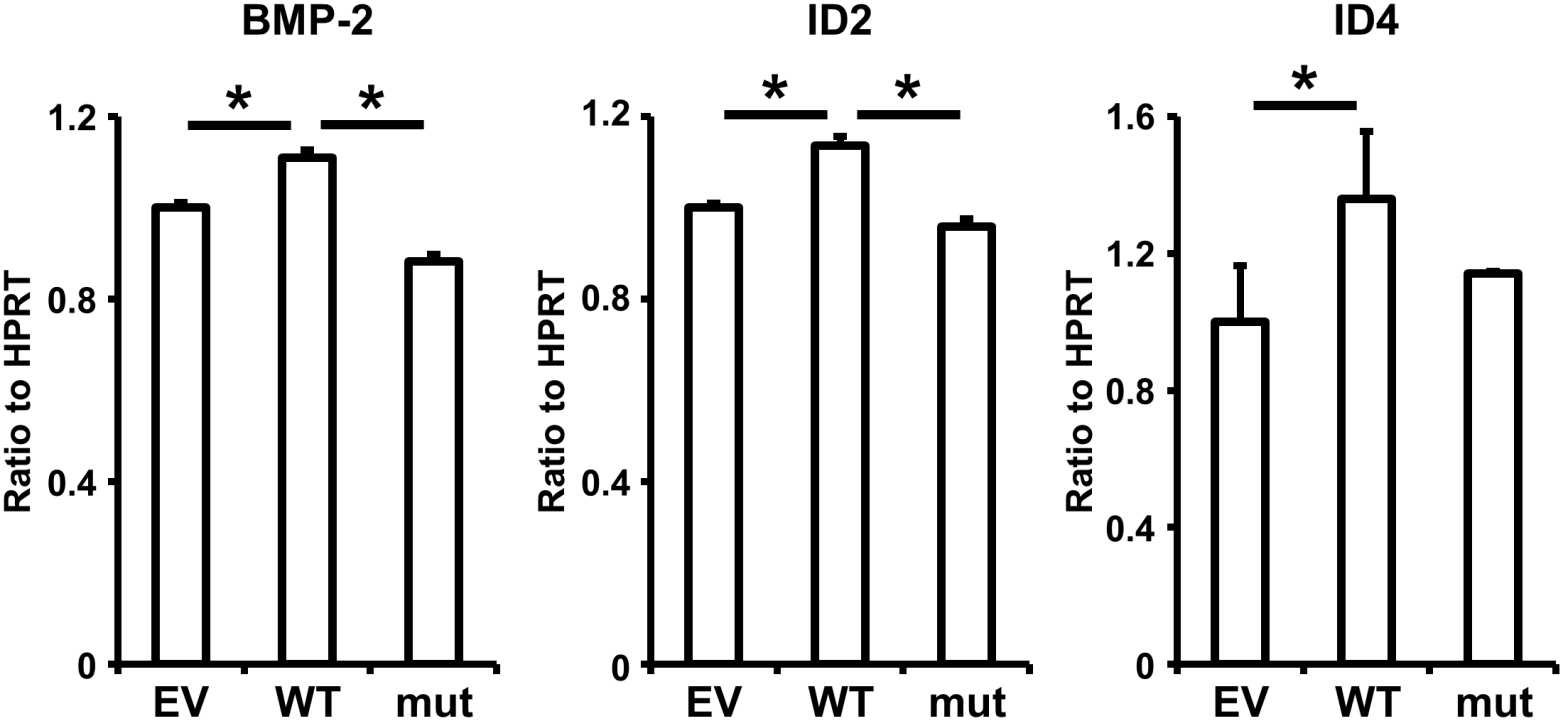
Effects of GPR126 on BMP-2, ID2, and ID4 expression in HPDL cells. HPDL cells were transfected with empty vector, wild-type GPR126, or mutant GPR126 for 96 h. The mRNA expression of BMP-2, ID2, and ID4 was assessed by real-time PCR. Data represent the average and standard deviation of three independent experiments. *p <0.05. EV, empty vector; WT, wild-type GPR126; mut, mutant GPR126.

GPR126 has been shown to elevate cAMP levels and PKA activation in Schwann cells of zebrafish and mice [41, 46]. The cAMP/PKA signaling pathway stimulates the osteoblastic differentiation of human mesenchymal stem cells via the induction of BMP-2, ID2, and ID4 expression [47]. To evaluate the effects of GPR126 on BMP-2, ID2, and ID4 expression in HPDL cells, wild-type or mutant GPR126 were introduced into HPDL cells and the expression of BMP-2, ID2, and ID4 were then assessed by real-time PCR. Wild-type GPR126 increased the mRNA expression of *BMP-2*, *ID2*, and *ID4*, whereas the mutant GPR126 had no effect on the expression (Fig 5). These data indicate that the GPR126-induced cytodifferentiation of HPDL cells depends on the induction of *BMP-2*, *ID2*, and *ID4* expression.

## Discussion

The present study utilized whole exome sequencing to identify novel genetic risk factors for AgP in a Japanese population. *GPR126* SNP rs536714306 showed a putative difference with respect to MAF between AgP cases and the Japanese control database HGVD. ELISA of cAMP concentrations indicated that the GPR126-induced cAMP/PKA signaling pathway was impaired in cells transfected with mutant GPR126 carrying rs536714306 compared with those carrying wild-type GPR126. Moreover, while GPR126 induced the cytodifferentiation of HPDL cells through the up-regulation of *BMP-2*, *ID2*, and *ID4* expression, rs536714306 had no effect on the cytodifferentiation of HPDL. Thus, GPR126 appears to be important in maintaining the homeostasis of periodontal tissue.

GWAS recently showed that a different genetic variant in *GPR126* [SNP rs6570507 (c.104-9134G>A)] is significantly associated with adolescent idiopathic scoliosis (AIS) in humans [48]. Affected patients have a three dimensional curvature (>10°) of the spine, and AIS is the most common form of spinal deformity in girls aged 10–16 years [49]. Several studies have shown that AIS patients have a low bone mineral density and the prevalence of AIS with osteopenia is 27–38% [50, 51]. *GPR126* knockout mice are smaller than their wild-type counterparts and display abnormal joint contractures of the forelimbs and hindlimbs [40], while *GPR126* knockout zebrafish have shorter body lengths and delayed ossification of the spine [48]. Moreover, a *GPR126* conditional deletion in cartilage tissues provides a mouse genetic model of AIS [52], indicating that GPR126 plays an important role in cytodifferentiation and the mineralization of osteoblasts and chondrocytes. Furthermore, signaling pathways such as BMP-2 [53], Wnt [37], and transforming growth factor-β [54], which regulate osteoblastic differentiation and endochondral ossification, also control cytodifferentiation and the mineralization of PDL cells. These findings strengthen our conclusion that GPR126 is important in regulating the cytodifferentiation of PDL cells.

If periodontitis is left untreated, it eventually leads to loss of affected teeth [42]. Thus, the ultimate goal of periodontal treatment is to regenerate the lost tissues. To induce periodontal regeneration, the effective regulation of PDL cell differentiation into osteoblasts and cementoblasts is important. The topical application of recombinant cytokines is one of the most effective regenerative procedures for enhancing osteoblastic differentiation. To date, however, the device using platelet-derived growth factor is the only commercially available product for periodontal regenerative therapy [55]. Because GPR126-induced elevation of cAMP enhanced the osteoblastic differentiation of PDL cells in our study, elevation of the cAMP/PKA signaling pathway may be a potentially important target to develop a novel periodontal regenerative therapy.

The present study had a number of limitations. Since its sample size is smaller than other conventional GWAS studies, our results lack genome-wide significance (*P*-value <5 × 10^−8^). We are now planning to collaborate with all dental universities in Japan to collect more patients with AgP, and thus obtain statistical results with genome-wide significance. Second, we lacked a replication study to confirm the association of the candidate gene with AgP [56]. To overcome this, however, we performed *in vitro* experiments to confirm the effects of the candidate gene in PDL cells. Despite these limitations, our study successfully used exome sequencing to identify a genetic variant of AgP. We also performed another *in vitro* functional study to analyze the expression of BMP-7 as assessed by real-time PCR. BMP-7 is known to enhance the cytodifferentiation of HPDL cells [57]. GPR126 significantly increased BMP-7 expression, whereas rs536714306 had no effects on it (data not shown). However, another study demonstrated that GPR126 mutant zebrafish showed greater expression of BMP-7 than did wild-type zebrafish in ear [58]. Further studies are necessary to understand this inconsistency.

Ideally, age- and sex-matched healthy controls would have been recruited to this study. However, because we currently lack appropriate control samples, we used HGVD as a controls, which were neither age- nor sex-matched with the AgP cases and have an unknown periodontal status. HGVD was previously used as a control database to identify genetic variants of retinitis pigmentosa [59]. While retinitis pigmentosa patients possessed the c.4957_4958insA variant with a MAF of 26.8%, no HGVD control subjects carried the variant. Thus, c.4957_4958insA was concluded as being important for the diagnosis of retinitis pigmentosa in the Japanese population. The use of HGVD controls in our study was deemed acceptable because the prevalence of AgP in the Japanese population is similar to that of retinitis pigmentosa, which is 1 in 3000–4000 [10]. Moreover, because the prevalence of AgP in the Japanese population is only 0.03% [10], it is reasonable to assume that the 729 HGVD controls would not suffer from AgP. It was also necessary to confirm that there are no shared genetic polymorphisms between chronic periodontitis (CP) and AgP. Although it is not clear whether genetic risk factors for CP and AgP are shared or different, Yasuda et al. previously described an association of Fc-gamma receptor genetic polymorphisms with both CP and AgP in a Japanese population [60]. However, this previous study demonstrated different genetic polymorphisms of the Fc-gamma receptor between CP and AgP, suggesting that shared genetic polymorphisms might not exist between CP and AgP.

GWAS offers a new opportunity to carry out an unbiased search for genetic variants of periodontitis [61]. GWAS has previously been used to identify genetic risk factors for chronic periodontitis, and several candidate genes were repeatedly detected in a similar population. For example, *NIN* was a suggestive gene in two western GWAS of a European American population and an American population [62–64]. More recently, *GPR141* was proposed as a suggestive gene in two Asian GWAS of Japanese and Korean populations [65, 66]. Interestingly, some genes identified in western GWAS were not detected in Asian GWAS, suggesting that differences in ethnic backgrounds may exist regarding genetic risk factors for chronic periodontitis.

In conclusion, our study indicates that *GPR126* SNP rs536714306 is a genetic variant for Japanese AgP. The SNP appeared to impair the signal transactivation of GPR126 and to have no effect on the cytodifferentiation of HPDL through increased *BMP-2*, *ID2*, and *ID4* expression. Although further national multicenter study is necessary to confirm that the GPR126 SNP is one of genetic risk factors for AgP in a Japanese population, these data could provide new insights into the diagnosis and treatment of AgP.

## Supporting Information

S1 Table List to show which teeth are affected with at least 30% bone loss for each patient. (DOCX)
