# Supplementary material for: A Putative Association of a Single Nucleotide Polymorphism in GPR126 with Aggressive Periodontitis in a Japanese Population
Source: PLoS One. 2016 Aug 10;11(8):e0160765. doi: 10.1371/journal.pone.0160765 (PMC4979892; doi:10.1371/journal.pone.0160765)
Supplement: S1 Table — (DOCX) [file pone.0160765.s001.docx]

**Appendix Table 1. List to show which teeth are affected with at least 30% bone loss for each patient**

Patient 1: 11, 12, 14, 15, 16, 23, 26, 27, 31, 36, 41, 42, 44, 45, 46

Patient 2: 11, 12, 13, 14, 15, 22, 23, 24, 25, 26, 27, 31, 42

Patient 3: 16, 17, 25, 26, 27, 28, 34, 35, 36, 37, 44, 45

Patient 4: 15, 16, 26, 27

Patient 5: 11, 16, 17, 21, 22, 25, 26, 27, 31, 35, 36, 37, 46, 47

Patient 6: 11, 12, 14, 15, 17, 21, 22, 23, 24, 25, 27, 31, 32, 34, 37, 41, 42, 45, 46, 47

Patient 7: 11, 12, 13, 14, 15, 16, 17, 21, 22, 23, 24, 25, 26, 27, 31, 33, 34, 35, 36, 37, 41, 43, 45, 46

Patient8: 11, 12, 13, 14, 15, 16, 17, 21, 23, 24, 25, 26, 27, 31, 32, 33, 34, 35, 36, 37, 41, 42, 43, 44, 45, 46, 47, 48

Patient 9: 11, 12, 13, 14, 15, 16, 17, 21, 22, 23, 24, 25, 26, 27, 34, 35, 36, 37, 43, 44, 46, 47

Patient 10: 11, 12, 13, 14, 15, 16, 21, 22, 23, 24, 25, 26, 31, 32, 33, 34, 35, 37, 41, 42, 43, 44

Patient 11: 11, 16, 17, 18, 21, 22, 25, 26, 27, 33, 34, 35, 36, 37, 43, 44, 46, 47

Patient 12: 12, 14, 15, 16, 17, 24, 26, 27, 35, 36, 37, 47

Patient 13: 11, 12, 13, 14, 21, 22, 25, 31, 32, 34, 35, 36, 41, 42, 43, 46

Patient 14: 11, 14, 16, 21, 23, 27, 31, 32, 33, 35, 36, 41, 42, 43, 45, 46

Patient 15: 16, 27, 37, 41

Patient 16: 11, 12, 14, 15, 16, 17, 21, 22, 24, 25, 26, 27, 31, 35, 36, 37, 42, 45, 46

Patient 17: 15, 16, 17, 26, 27, 35, 36, 37, 42, 44, 45, 46, 47

Patient 18: 16, 17, 18, 21, 25, 27, 28, 32, 33, 34, 35, 36, 37, 38, 41, 42, 43, 46, 47

Patient 19: 12, 16, 21, 22, 23, 26, 27, 36, 42, 43, 46

Patient 20: 11, 12, 13, 16, 17, 24, 25, 26, 27, 32, 33, 41, 42, 45

Patient 21: 13, 15, 16, 21, 22, 26, 28, 31, 32, 36, 41, 42, 46, 47

Patient 22: 11, 12, 13, 14, 16, 17, 21, 22, 23, 24, 26, 27, 31, 32, 33, 34, 36, 37, 41, 42, 43, 45, 46, 47

Patient 23: 11, 12, 13, 14, 15, 16, 17, 21, 22, 23, 24, 25, 26, 27, 31, 32, 33, 34, 35, 36, 37, 41, 42, 43, 44, 45, 46, 47

Patient 24: 11, 12, 13, 14, 15, 16, 17, 21, 22, 23, 25, 26, 27, 31, 32, 33, 34, 35, 36, 37, 41, 42, 43, 44, 45, 46, 47

Patient 25: 11, 12, 14, 15, 16, 17, 21, 22, 23, 24, 25, 26, 27, 31, 32, 33, 34, 35, 36, 37, 41, 42, 43, 44, 45, 46, 47

Patient 26: 11, 12, 15, 16, 21, 22, 31, 34, 41, 42, 43, 44

Patient 27: 13, 14, 15, 16, 17, 18, 22, 23, 24, 25, 26, 27, 31, 33, 34, 35, 36, 37, 41, 44, 45, 46, 47

Patient 28: 11, 15, 16, 17, 22, 23, 24, 27, 28, 31, 32, 33, 34, 35, 36, 37, 38, 41, 43, 44, 45, 46

Patient 29: 11, 12, 13, 14, 15, 16, 17, 21, 22, 23, 25, 26, 27, 31, 34, 36, 37, 38, 41, 43, 44, 45, 46

Patient 30: 11, 13, 14, 15, 16, 17, 22, 24, 25, 26, 27, 31, 32, 33, 34, 35, 41, 42, 43, 44, 45

Patient 31: 12, 13, 14, 15, 16, 17, 18, 22, 24, 25, 26, 27, 33, 34, 35, 36, 37, 38, 43, 44, 45, 46, 48

Patient 32: 11, 12, 15, 16, 21, 22, 23, 25, 26, 27, 31, 32, 33, 34, 35, 41, 42, 43, 45, 46, 47

Patient 33: 25, 32, 33, 34, 36, 41, 47

Patient 34: 14, 15, 16, 17, 25, 26, 27, 32, 33, 34, 36, 37, 47

Patient 35: 12, 13, 16, 26, 27, 31, 34, 42, 43

Patient 36: 11, 16, 21, 22, 26, 31, 32, 36, 41, 42

Patient 37: 17, 24, 31, 37, 41

Patient 38: 11, 12, 13, 16, 17, 18, 21, 22, 23, 24, 25, 26, 27, 31, 32, 36, 37, 41, 42, 43, 44, 46, 47

Patient 39: 15, 16, 22, 25, 26, 45

Patient 40: 11, 12, 14, 15, 16, 21, 22, 25, 26, 31, 32, 33, 34, 35, 36, 37, 38, 41, 42, 43, 44, 45, 46

Patient 41: 11, 12, 13, 14, 15, 17, 21, 22, 23, 24, 26, 27, 34, 36, 46, 47
